# Convergent adaptive evolution—how common, or how rare?

**DOI:** 10.1093/nsr/nwaa081

**Published:** 2020-05-12

**Authors:** Chung-I Wu, Guo-Dong Wang, Shuhua Xu

**Affiliations:** School of Life Sciences, Sun Yat-Sen University, China; Kunming Institute of Zoology, Chinese Academy of Sciences, China; Center for Excellence in Animal Evolution and Genetics, Chinese Academy of Sciences, China; Center for Excellence in Animal Evolution and Genetics, Chinese Academy of Sciences, China; CAS-MPG Partner Institute for Computational Biology, Shanghai Institutes for Biological Sciences, Chinese Academy of Sciences, China

This Special Topic contains one perspective and three original articles that address the topic of convergent evolution. How common, and how important, is convergent adaptation in evolution? These are the questions these studies attempt to answer.

If the concept of evolution has to be encapsulated in a single word, that word must be adaptation or, in a few more words, selection for the fittest. Nevertheless, proving adaptation is extremely difficult. For example, if human intelligence is adaptive (as we suspect that it must be), why are *Homo sapiens* the only species sufficiently intelligent to contemplate its own adaptation? One way to show adaptation, dating back to Darwin's *On the Origin of Species*, is convergent evolution. When multiple taxa independently evolved the same trait to cope with the same, or similar, environmental pressures, the trait is likely adaptive in that environment.

There are two aspects to the concept—convergent evolution and parallel evolution. When a new environment demands a new phenotypic state A (such as low-oxygen tolerance in high altitude), two species that evolve from the old phenotypic state of B and C, respectively, are said to have undergone convergent evolution. If they both evolved from B to A, then it is parallel evolution. When dealing with genomic sequences, the distinction would lose its usefulness, as the separate evolution from B to A and from C to A, is too common to be meaningfully analyzed. Here, the term convergent evolution is used for multiple, independent evolution from B to A.

As each paper in this issue shows, convergent evolution at the phenotypic level is rather common. In Zhang *et al*., seahorses, pipefishes and seadragons all have tubular mouths and male pregnancy [1]. In Wu *et al*., most domesticated mammals colonizing the Tibetan plateau exhibit a host of traits pertaining to the tolerance of low oxygen, high UV and daily temperature swings [2]. For woody plants that colonize the interface between the land and sea (known collectively as mangroves), He *et al*. present the convergence in salt tolerance, aerial roots and vivipary [3].

All four papers address the molecular basis of convergent evolution. In other words, they attempt to understand the genetic wiring underneath the adaptation to extreme environments. The same genetic wiring may truly reflect the converging evolutionary mechanisms beyond the appearance of similar adaptations. Genetic convergence can happen at several levels—at a specific site of a gene, at any site of the same gene, along the same genetic pathway, or in the structure of genes, genomes, or simply their expressions. Together, these four studies cover many of these levels, thus broadening the concept of genetic convergence.

At its most stringent, the concept of convergence means that the species have the same amino acid change at the same site of the same gene. He *et al*.’s perspective makes an important but surprising point [4]. They find no convincing evidence for *‘*site convergence*’* in the literature of genomic surveys. The reason is that the level of background convergence in the control group is usually as high as that in the focus group. Indeed, earlier studies have repeatedly shown that convergent evolution is a process with very high noise/signal ratio, rendering previous conclusions of true convergence suspect [5].

Following up the extensive surveys, He *et al*. propose a semi-quantitative criterion for determining molecular convergence [3]. Like all evolutionary analyses, the inference of convergence is probabilistic and, if at all possible, this probability should be presented to provide some guidance to further *‘*functional*’* analyses. Using a symmetrically placed control group, they identify 73 genes of true convergence with a probability of >0.9 among mangroves. In Zhang *et al*., heeding the warning of high noise/signal ratios, the authors winnow the candidate gene list down to a few that may control pregnancy in both placental mammals and seahorses/pipefish. Again, genes on this list are candidate genes that await functional validation but the analysis has provided valuable clues.

In Wu *et al*., a total of 327 genomes were analyzed for the identification of the divergence between domesticated mammals of highlands and low altitude [2]. The study inspects each gene in its entirety for signals of adaptive evolution on the Tibetan plateau. The approach is closest to the efforts to identify the common causes of tumorigenesis, championed by the Cancer Genome Atlas (TCGA) [6,7]. If a gene is important for high-altitude adaptation or cell proliferation, it probably has multiple sites that can effect the adaptation. While the logic seems reasonable, TCGA has found that the same pathologically defined cancers often have mutations in totally different sets of genes [6,8]. Note that TCGA has surveyed, in total, more than ten thousand genomes, far more than in Wu *et al*.’s efforts; the latter nevertheless have identified a few genes of convergence.

Why would the same approach find very different degrees of convergent evolution? There are fragments of answers to this question. A piece of the puzzle is the number of genetic solutions available for attaining the new phenotype [9,10]. There may be a great number of genetic pathways for cells to proliferate rapidly. In that case, genetic convergence is not expected in tumorigenesis. In contrast, Wu *et al*.’s results appear to suggest that mutations of EPAS1 might be particularly effective in coping with the adaptation on the Tibetan plateau. Hence, genetic solutions devised by mammals often involve this gene. The analyses also open up many more avenues of inquiry. For example, why did the adaptation on the Ethiopian highland or in the Andes mountains not go through EPAS 1 [11,12]? By asking such questions, one may be able to develop hypotheses on the likelihood of convergence, prior to carrying out the empirical studies.

In a higher level of convergence, He *et al*. also show that mangroves converge in genome-wide amino acid usages [3]. For the adaptation in the interface between land and sea, woody plants have evolved different usages of amino acids that are shared among mangroves, and only mangroves. A forthcoming NSR publication on plant secondary metabolites further highlights the phenotypic convergence in the apparent absence of genetic convergence, even when the phenotypes are biochemical in nature [13]. Overall, these publications find various degrees of genetic convergence in similar environments. Collectively, molecular convergence is much more broadly defined than it has been in the literature.

Whether convergent adaptive evolution is common or rare, as asked in the title of this commentary, may depend on the level of the genetic hierarchy one is interested in. Nevertheless, a conclusion of adaptive convergent evolution is justified, regardless of the level of the genetic hierarchy in which the convergence is observed. Finally, while convergence inferences by evolutionary analysis may not be considered functional evidence, they can often be effective in identifying promising candidate genes, sometimes more effective than the strictly functional considerations. In the case of neoteny in human development, a good candidate gene emerges when it shows a strong evolutionary signature and has indeed been experimentally validated [14]. These publications are following a similar intellectual pursuit.
